# Drug repositioning in sarcomas and other rare tumors

**DOI:** 10.1016/j.ebiom.2016.03.021

**Published:** 2016-03-17

**Authors:** Alex T.J. Lee, Paul H. Huang, Seth M. Pollack, Robin L. Jones

**Affiliations:** a Sarcoma Unit, Royal Marsden NHS Foundation Trust, London, UK; b Institute of Cancer Research, London, UK; c Fred Hutchinson Cancer Research Center, Seattle, WA, USA; d University of Washington, Seattle, WA, USA

**Keywords:** Drug repositioning, Drug development, Sarcomas, Angiosarcoma

The exploration and identification of new anti-cancer therapeutic indications for an approved drug already in use, or for a drug that was shelved for non-safety reasons in early clinical development, represents an attractive alternative to the costly, time-consuming and inefficient process of developing novel anticancer agents. Drug repositioning or repurposing can bypass the discovery and early clinical phases of cancer drug development, potentially reducing the hundreds of millions of dollars and more than a decade typically required to see a novel agent through to regulatory approval. Incidental clinical observations, case–control analysis of large populations and modern high-throughput drug screening techniques have been employed in identifying agents with hitherto undiscovered or ignored biological activity that could underpin effective anticancer activity.

To date, the most significant example of drug repositioning in oncology has seen thalidomide and its analog lenalidomide approved for the treatment of multiple myeloma.(Engelhardt et al., 2014) Notoriously developed as a sedative and anti-emetic in the 1950s, thalidomide was decades later seen to have anti-angiogenic effects in animal models, research that translated into significant clinical activity in myeloma, acute myeloid leukemia and myelodysplastic syndrome. Large scale prospective studies of aspirin chemoprophylaxis in the primary or secondary prevention of several common cancers are underway with the goal of confirming recurring evidence of effect detected by retrospective analysis of large cardiovascular study cohorts (Rothwell et al., 2011). Similarly, the association in several epidemiological studies of use of the widely-prescribed diabetes drug metformin with reduced cancer incidence and mortality has prompted a great deal of ongoing preclinical and clinical investigation of the drug's apparent anticancer effect.(Quinn et al., 2013)

Sarcomas are rare tumors of mesenchymal origin, encompassing over 70 histological subtypes of distinct clinical behavior and underlying biology. Metastatic disease is common and associated with poor prognosis. Diverse molecular pathology and, in many cases, lack of effective systemic therapies indicate a compelling case for drug repurposing studies in sarcoma. The characterization of recurrent mutations of cKIT in gastrointestinal stromal tumors (GIST) and TSC1/2 in malignant peripheral epithelioid cell tumors (PEComa) formed the basis for the respective repurposing of imatinib and sirolimus. Imatinib, a small molecule inhibitor originally developed to target BCR-ABL in chronic myeloid leukemia but whose kinase inhibition extends to cKIT, has dramatically improved survival outcomes in both early-stage and advanced GIST,(Rajendra et al., 2013) whilst unprecedented response rates were seen in PEComa in case series treated with sirolimus, an inhibitor of the mTOR complex used in the prevention of organ transplant rejection.(Benson et al., 2014)

Propranolol has been successfully repurposed for the treatment of infantile hemangioma, based on a number of initial clinical observations and a subsequent randomized trial.(Léauté-Labrèze et al., 2015) Furthermore, a number of pre-clinical and clinical studies have indicated that beta-blockade may have a potential therapeutic role in the management of various cancers, including angiosarcoma.(Chow et al., 2015) Pasquier and colleagues previously demonstrated that the addition of beta blocker enhanced anti-proliferative and anti-angiogenic effects of co-administered cytotoxics in animal models of breast cancer and neuroblastoma.(Pasquier et al., 2011, Pasquier et al., 2013) In this issue of *EBioMedicine*, they now describe their retrospective evaluation of a combination of propranolol and vinblastine-based chemotherapy in a small series of patients with metastatic angiosarcoma.(Pasquier et al., 2016) These data, although retrospective and provisional, are worthy of further prospective evaluation and highlight the possibility of drug re-positioning in rare tumors such as sarcoma. The Food and Drug Administration orphan drug designation was proposed as a means to reduce the development costs of therapy in rare diseases (< 200,000 patients in the United States), and this may be an avenue for drug repositioning. Vigilant clinical observation and reporting in combination with *in vitro* and *in silico* drug screening that exploits a burgeoning understanding of sarcoma molecular pathology can be employed to hopefully identify drugs that are candidates for repositioning studies.

The study raises a number of relevant questions regarding research and drug development in rare cancers. Obtaining access to drugs such as propranolol for a small number of patients is relatively easy, but performing a randomized trial in a very rare and heterogeneous entity such as angiosarcoma is a considerable financial and logistical challenge. Large international multicenter studies are required to achieve sufficient recruitment, a considerable undertaking that pharmaceutical companies are unlikely to consider due to the lack of return from generically available products. There is, however, a strong track record of investigator-initiated trials within the international sarcoma community. The funding of such trials by funding agencies or charities may be limited, as such projects may be deemed less exciting or innovative compared with other studies evaluating a novel agent. In addition, drug repositioning is not risk free, and involves the use of a drug in a new patient population with potentially differing pathophysiological characteristics and also new drug formulations and dosing schedules.

In conclusion, the study by Pasquier and colleagues is of interest and should be explored further in a prospective trial. Drug repositioning could potentially be of benefit in other sarcoma subtypes particularly those with currently no effective systemic therapy. Such an approach clearly requires collaboration between basic, translational and clinical researchers.

## Disclosure

The authors declared no conflicts of interest.
